# Analysis of the Innovation Trend in Cell-Free Synthetic Biology

**DOI:** 10.3390/life11060551

**Published:** 2021-06-11

**Authors:** Conary Meyer, Yusuke Nakamura, Blake J. Rasor, Ashty S. Karim, Michael C. Jewett, Cheemeng Tan

**Affiliations:** 1 Department of Biomedical Engineering, University of California, Davis, CA 95618, USA; conmeyer@ucdavis.edu (C.M.); triom812@gmail.com (Y.N.); 2 Department of Chemical and Biological Engineering and Center for Synthetic Biology, Northwestern University, Evanston, IL 60208, USA; blakerasor2023@u.northwestern.edu (B.J.R.); ashty.karim@northwestern.edu (A.S.K.); m-jewett@northwestern.edu (M.C.J.)

**Keywords:** synthetic biology, cell-free protein synthesis, TX-TL, transcription and translation, patent, industry

## Abstract

Cell-free synthetic biology is a maturing field that aims to assemble biomolecular reactions outside cells for compelling applications in drug discovery, metabolic engineering, biomanufacturing, diagnostics, and education. Cell-free systems have several key features. They circumvent mechanisms that have evolved to facilitate species survival, bypass limitations on molecular transport across the cell wall, enable high-yielding and rapid synthesis of proteins without creating recombinant cells, and provide high tolerance towards toxic substrates or products. Here, we analyze ~750 published patents and ~2000 peer-reviewed manuscripts in the field of cell-free systems. Three hallmarks emerged. First, we found that both patent filings and manuscript publications per year are significantly increasing (five-fold and 1.5-fold over the last decade, respectively). Second, we observed that the innovation landscape has changed. Patent applications were dominated by Japan in the early 2000s before shifting to China and the USA in recent years. Finally, we discovered an increasing prevalence of biotechnology companies using cell-free systems. Our analysis has broad implications on the future development of cell-free synthetic biology for commercial and industrial applications.

## 1. Introduction

**Figure 1:**
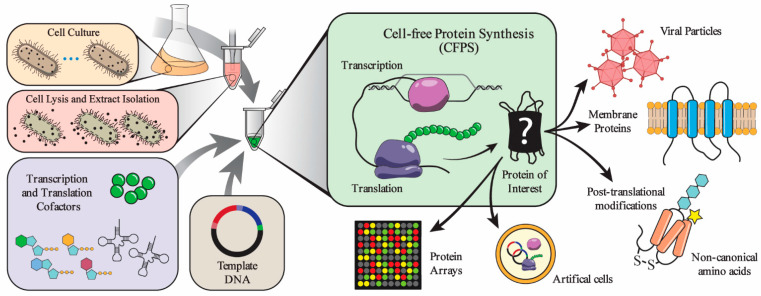
The components and applications of cell-free protein synthesis (CFPS). CFPS systems decouple the production of proteins from cell viability, which allows for a highly tunable system that can be used for on-demand protein synthesis in a variety of environments to produce a broad spectrum of proteins. A major component of a CFPS system is the protein expression machinery. The machinery includes RNA polymerases, ribosomes, translation factors, etc. These proteins generally come from whole-cell extracts. Whole-cell extracts are produced by culturing the cell type of interest, lysing the cells, and extracting the cellular components. The proteins can also be acquired through protein production and purification. The other major set of components are the transcription and translation cofactors and reactants. These include tRNAs, rNTPs, amino acids, etc.. The mixture of the protein and small molecule solutions can then be used to make a desired protein or collection of proteins with the addition of a DNA sequence. The DNA is transcribed by an RNA polymerase, and then the ribosome translates the resulting RNA with the help of the translation factors and aminoacyl-tRNA synthetases to produce the protein of interest. The DNA can encode the sequence for desirable proteins such as membrane proteins or collections of proteins to make viral particles. CFPS can also be used to make proteins with modifications, such as disulfide bonds, glycosylation, and non-canonical amino acids. CFPS systems can be assembled in different environments, such as inside liposomes to create artificial cells or be aliquoted into microarrays for protein screening.

In 1961, Heinrich Matthaei and Marshall Nirenberg used *Escherichia coli* S30 extract to decipher the genetic code, which later earned them the 1968 Nobel prize in Physiology or Medicine [1]. Since then, cell-free protein synthesis (CFPS) has enabled the engineering and study of cellular processes without using living cells [2,3,4,5,6,7,8]. To achieve CFPS, cellular machinery for transcription, translation, and energy regeneration is extracted from cells, then assembled with reaction components in a range of reaction vessel types, including well-plates [9], droplets [10], or liposomes [11] (Figure 1). CFPS offers several advantages over other methods. First, the open nature of the reaction allows the user to directly influence the biochemical systems of interest. As a result, new components can be added or synthesized, and these can be maintained at precise concentrations. This can include, for example, non-canonical amino acids for incorporation into proteins that expand the chemistry of life. Second, the chemical environment can be controlled, actively monitored, and rapidly sampled. Third, processes that take days or weeks to design, prepare, and execute in cells can be done more rapidly in a cell-free system, since no time-consuming cloning steps are needed and reactions are easily automated [5,8,12].

Despite the advantages, CFPS still has challenges to widespread commercial adoption including costs, enzyme stability, reaction duration, inefficiency of some post-translational modifications, and unfamiliarity with the technology [5]. Recent work has attempted to overcome these challenges. For instance, *E. coli* strains were modified to enhance protein synthesis yield and reduce production costs [13]. Furthermore, protein synthesis machinery was enhanced to synthesize proteins with glycosylation and non-canonical amino-acids [14,15,16,17]. In addition, the preparation process of CFPS was simplified and streamlined to minimize cost and batch variability [18,19]. These recent innovations have boosted the commercial interest in CFPS. Several companies now sell CFPS kits, including Life Technologies, Promega, Arbor Biosciences, Thermo Scientific, WEPRO, Qiagen, Lenio Bio, New England Bio Labs, and GeneFrontier, among others [20]. There are also increasing interests in using CFPS to produce commercial therapeutic proteins, synthesize glycoproteins, prototype cellular pathways, enable portable molecular diagnostics, and synthesize virus-like particles as vaccines [8,17,21,22,23] (Figure 1). Recent papers have reviewed the history of CFPS and its myriad of applications [4,5,24].

Based on the recent and rapid development in CFPS, what can we expect for the future of cell-free synthetic biology? Can we learn lessons from the field’s history to guide future development? For instance, which areas of innovation usher in new CFPS-based applications? Answers to these questions may help us prioritize the next investments in cell-free systems both in academia and industry. Toward these answers, patents are often used as the indicators for future commercial applications or investment activity. Patent analysis has been invaluable in clarifying the trend and magnitude of technology development [25]. However, patent and publication trends are unable to fully capture the exact state of research in a given field as the underlying economic, scientific, and societal factors coalesce into the observed output of papers and patents. Here, we cataloged patent publications and peer-reviewed manuscripts relating to CFPS from public databases. We then curated these publications by subject matter, applicants/authors, and countries. Finally, we analyzed the type of innovations that spurred new waves of development in cell-free systems.

## 2. Materials and Methods

M1. Generation of the patent and publication database: To ensure the acquisition of both patents and publications was inclusive of all those around the world, global repositories were used to pull the data. For the patent searches, the World Intellectual Property Organization’s (WIPO) PATENTSCOPE search engine was used [26]. The cross-lingual expansion search was used to expand all search terms to all languages to ensure that coverage of patents from all countries was equitably returned. The expansion mode was set to ‘Automatic’ and the precision level was set to ‘high’. For the publication search, the Scopus database and search engine were used [27]. The search criteria covered the title, abstract, and keywords. Both the patent and publication searches used the same search terms. The included terms and number of returned documents are as follows: “cell-free protein synthesis” (patents-677, publications-1171), “cell-free synthesis” (patents-2357, publications-297), “cell-free translation” (patents-2909, publications-1096), “in vitro protein synthesis” (patents-473, publications-321), “in vitro protein translation” (patents-307, publications-28). All searches were restricted to the time frame of 1990 to 2020. The searches were conducted on January 25th, 2021.

M2. Curation and categorization of patents and publications: Curation of the patents and publications included elimination of duplicates of documents that shared the same title. Then documents missing publication dates or authors were removed. After these steps, 5225 patents and 2637 publications remained. The patent data was further curated by restricting the patents to those filed by the following countries: World-wide (WO), United States (US), Japan (JP), Europe (EP), China (CN), and Canada (CA). Patents from these countries included 2098 patents. Publications were assigned countries based on the affiliation of the corresponding author. These publications were manually categorized into the following categories: Improve CFPS, Protein modification, Specific proteins, and Applications. In both the patent and publication search results, there were numerous documents that were returned that did not pertain to the technology discussed in this article. Numerous patents were removed as they referred to topics including cancer therapeutics and fuel cells. The eliminated publications included the use of a cell-free protein synthesis assay where cells were lysed and tested for inhibition of protein synthesis, mostly for the study of antibiotics. Review articles were also eliminated. This curation left 762 patents and 1723 publications that were relevant to the CFPS field. The applicants of the patents were also curated to group the applicants regardless of the variation of the name used. All the patents and publications used in this study, including all metadata and categorization, are available in the Supplementary Data File.

## 3. Results

### 3.1. Patent and Publication Counts Are Trending Positively from 2010 to Present

**Figure 2:**
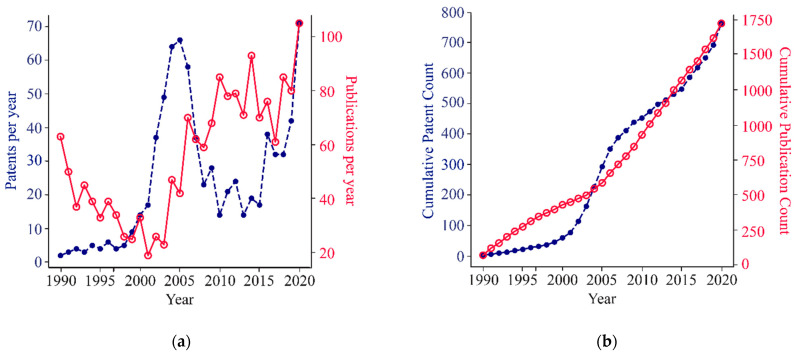
Fluctuation in the number of patent and publications over time. Patent filings per year increased by more than 4-fold between 2015 and 2020. From 2003 to 2020, there is a 5-fold increase in publications. Patents (blue dotted line), n = 762; Publications (red full line), n = 1723. See Methods M1 and M2 for details on data source. (**a**) Patents and manuscripts published per year; (**b**) Cumulative total of all patents and publications over time. See Methods M1 and M2 for details on data source.

To investigate whether the recent interest in CFPS systems is backed by tangible progress, the total counts of patents and publications were calculated over the period of 1990 to 2020 (Figure 2a,b). In the patent trace, there is a roughly seven-year spike in patents centered around 2005, where the number of filings per year increased by 12-fold compared to the rate at the beginning of the spike. This spike is followed by a steady period of a few filings per year and a sharp rise after 2015 with a four-fold increase in filings per year by 2020. Conversely, CFPS innovations were documented more prominently in published, peer-reviewed manuscripts in the early 1990s, with over 400 manuscripts published during that period. At the cusp of the mid-2000s patent spike, published manuscripts were down to 19 per year in 2001. After the turn of the 21st century and the dawn of the synthetic biology era [28], we see a dramatic five-fold increase in publication rate reaching over 100 publications per year. The rise in publications following the peak in patents is possibly explained by the increased availability of commercial products, such as CFPS kits. These products could have increased access to the technology and allowed more labs to innovate with cell-free technologies. Future work would need to systematically evaluate the use of patented technology in the publications that followed the patent spike to assess if the rise in publications after the rise in patents is causal. Of note, the field has had multiple definitions, which have included cell-free translation, cell-free protein synthesis (CFPS), in vitro transcription and translation (IVTT), TX-TL, and cell-free gene expression (CFE), among others. The different naming conventions provide a challenge to assessing the true impact and reach of the field. All patents and publications used in this study are available in the Supplementary Data File.

### 3.2. Patents and Publications Are Increasing across a Broad Range of Sub-Topics

Investigating the types of advances being made across the patent and publication landscape is essential to understanding innovation trends in cell-free synthetic biology. To do this, we manually categorized all documents into subtopics: ‘Improved CFPS’, ‘Protein Modifications’, ‘Specific Proteins’, and ‘Applications’ (refer to Methods section M2). The ‘Improved CFPS’ category includes system-level improvements of CFPS (e.g., reagents, processing, operation). These improvements can include increasing accessibility to the technology with commercial CFPS kits, generation of extracts from non-model organisms, such as wheat-germ [29] and Xenopus [30], for improved functionality, and methods to optimize protein synthesis yields such as modifying amino acid metabolism in bacterial extracts [31]. The ‘Protein Modifications’ category includes the alteration of CFPS systems to include mechanisms for post-translational modifications (e.g., disulfide bonds [32], glycosylation [33], etc.) and incorporation of non-canonical amino acids [34]. These modifications can allow for increased functionality of the protein by incorporating new chemical moieties in non-canonical amino acids for non-natural site-specific chemistry or fluorescent labelling. ‘Specific proteins’ include methods utilizing CFPS to make proteins that are otherwise challenging to make. These proteins include therapeutic [23,35] and membrane proteins [36], among others. ‘Applications’ include microfluidic systems for protein arrays using CFPS [37], incorporation into artificial cells [11], and kits for paper-based detection of pathogens or small molecules [38].

**Figure 3:**
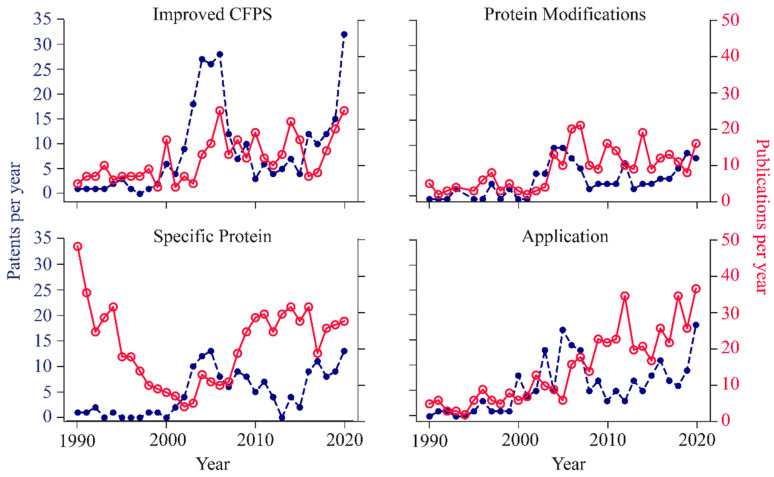
Fluctuations in patents and publications pertaining to specific topics. Publications pertaining to synthesis of specific proteins accounted for 63% of all publications between 1990 and 2000. Patents pertaining to improving CFPS systems accounted for 41% of all patents published between 2001 and 2008. The patents published between 2001 and 2008 made up 45% of all patents published between 1990 and 2020. From 2015 to 2020 the average fold increase in patents published per year across all categories is 5-fold. Patents are plotted as blue dashed lines and publications are plotted as red solid lines. Top-left: ‘Improved CFPS’, n = 270 patents, 365 publications. Top-right: ‘Protein Modifications’, n = 140 patents, 271 publications. Bottom-left: ‘Specific Protein’, n = 151 patents, 659 publications. Bottom-right: ‘Application’, n = 182 patents, 428 publications.

In the 1990s, nearly all the publications pertained to the synthesis of ‘specific proteins’ (Figure 3). These early papers primarily used CFPS systems to synthesize viral particles for their study in vitro. Over the 30 year period between 1990 and 2020, researchers transitioned their focus toward membrane-bound proteins, protein libraries, therapeutics, diagnostics, and improving cell-free protein synthesis systems. While ‘specific protein’ synthesis has dominated the publication space for CFPS, even this category saw a 10-fold drop from 1990 to 2003 when patents pertaining to the synthesis of ‘specific proteins’, as well as CFPS patents more broadly, were on the rise. After the 2005 patent spike and as CFPS was becoming more accessible (commercially available), we observed the most abrupt increase in publications of CFPS of ‘specific proteins’. However, from 2015 to 2020 we have seen an increase in CFPS publications across all categories with a steady increase in growth of the CFPS ‘applications’ space.

On the other hand, the patent space has seen less dramatic changes over time, excluding the patent spike of 2005, and potential recent rise. While very few patents were filed in the 1990s, most of the early patents were focused on improving CFPS. Looking closely, more than 40% of all patents filed during the 2001–2008 ‘patent spike’ pertained to ‘Improved CFPS’ (Figure 3), with a key focus on extending reaction durations, reducing costs, and enhancing functionality. Creating enhanced systems for performing CFPS, likely increased accessibility and interest to innovate in the other categories. After the spike period, all categories saw a steady ~5 patents per year between 2008 and 2015. Between 2015 and 2020, we see steady growth in patents in all categories, averaging ~3 additional patents per category per year. This happens to coincide with both the rise in CRISPR innovations and the historically low cost for DNA synthesis. While these do not explain all CFPS innovation, they provide contextual understanding of this emerging golden age of cell-free synthetic biology.

### 3.3. Volatility in Patent Filings Is Driven by Commercial Entities

**Figure 4:**
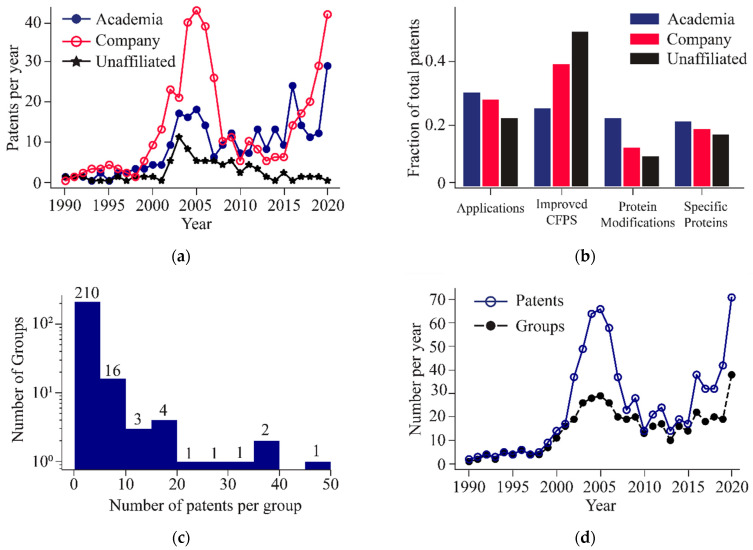
Breakdown of the patent filers. Patents published by companies account for 61% of all patents published between 2001 and 2008 and 55% between 2015 and 2020. Entities that filed a single patent comprises 55% of all entities, and entities that filed less than 5 patents accounts for 88%. In 2005 there is a 2.3-fold difference between the total number of patents filed and the number of unique filers and 2020 there is a 1.9-fold difference. (**a**) Plot of total number of patents published per year per group type, including companies (n = 421, red line), academic institutions (n = 271, blue line), and unaffiliated individuals (n = 64, black line). (**b**) Chart of the fraction of topic areas that each type of group patented on. (**c**) Histogram of the number of companies that have filed a specific number of patents (n = 239). (**d**) Plot showing the total count of patents (n = 762, blue line) and the number of individual groups filing per year (n = 456 black dashed line).

To better understand how innovation has changed over time, we categorized patent innovation into three groups based on affiliations of patent filers: Academia (university and government labs), Company, and Unaffiliated. Companies appear to have driven the ‘patent spike’ we see in the mid-2000s (Figure 4a). From 2000 to 2005, patents filed by companies accounted for 60% of all patents. By 2008, company-based filings had dropped and remained at around eight filings per year until 2015. Renewed commercial interest in CFPS systems is evident by the abrupt seven-fold increase in company-based patent filings from 2015 to 2020. Filings by academic and unaffiliated groups showed an increase of about five filings per year from 2001–2003 like the rate of company filings. However, both categories peaked at less than half the total filings from companies. Filings in academia have slowly trended upward at an average rate of one additional patent per year from 2007 to 2020, resulting in a five-fold increase in patent publications. Patents published by unaffiliated groups have remained roughly constant. The topic of patents filed by academic groups was distributed within a 10% range among the four categories (Figure 4b). Unaffiliated groups, however, showed a heavy focus on ‘Improved CFPS’ patents, accounting for 50% of all patents filed by this group type. Companies also favored ‘Improved CFPS’ as 40% of all patents filed by companies focused on this subject.

We next sought to reveal whether a handful of entities dominated CFPS developments or if innovation was more distributed. To do this, we documented the number of patent filings per group over the 30-year period from 1990 to 2020. We found that more than half of all groups filed a single patent, with 95% of all groups filing less than 10 patents (Figure 4c). While many groups have participated in advancing the field, there are a handful that have significantly contributed to the innovating CFPS. Notably, four groups contributed more than 30 patents each. These groups are Kangma Biological Technology CO., Cell Free Sciences Co., Ltd., Shimadzu Corporation, and Riken. The academic institutions with the most filings are Northwestern University, Stanford University, and the University of Tokyo, each filing 18 patents between 1990 and 2020. Furthermore, we plotted the number of unique groups filing each year (Figure 4d). During the spike from 2001 to 2007, and after 2015, there is a clear divergence between the number of patents and unique groups, with the average number of patents per group more than doubling. Outside of these ranges, groups were generally publishing a small number of patents each.

### 3.4. Advances in Cell-Free Synthetic Biology Continue to Expand Industrial Applications

The current growth of both commercial and academic patents correlates with the diversification of cell-free systems beyond the production of ‘specific proteins’ that dominated early commercial efforts. However, therapeutic protein production maintains significant commercial interest as researchers continue to optimize post-translational modifications, protein yields, and reaction scales of cell-free systems [39,40]. The flexibility and efficiency of CFPS also enable commercial ventures beyond the traditional mass production and delivery pipeline in the form of distributed, or decentralized, production of bioactive molecules, including conjugate vaccines and antibody analogs [17,41]. Furthermore, distributed cell-free synthetic biology has demonstrated value for point-of-use testing for pathogens and water contaminants [21,42] as well as inexpensive biology education platforms [43,44]. CFPS applications also continue to grow for discovering and optimizing enzymes to make commodity chemicals [45], natural products [46], and biomaterials [47], although price and scalability remain significant hurdles to industrial production volumes [7]. Meanwhile, highly optimized, small-scale CFPS platforms can reduce design-build-test cycles for increased commercial productivity by rapidly screening enzyme and therapeutic libraries [22,48,49]. Commercial interest in each of these areas suggests that growth will continue in the number of companies patenting cell-free technologies, particularly as start-ups continue to form and be acquired.

### 3.5. Recent Increase in the Number of Patents and Publications Are Driven by the United States, Europe, and China

**Figure 5:**
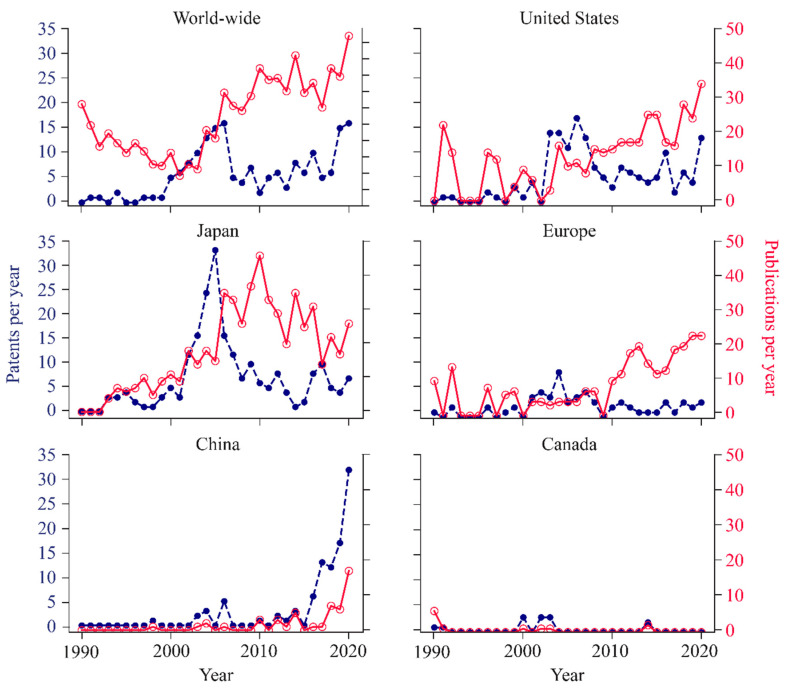
Breakdown of the number of patents and manuscripts published by region. Between 2001 and 2008, patents published by Japan accounted for 37% of the total patents for that time-period, United States accounted for 24% and world-wide patents accounted for 23%. Between 2015 and 2020, patents published by China accounted for 35% of the total patents for that time-period, United States accounted for 17% and world-wide patents accounted for 26%. Between 2015 and 2020, manuscripts published by the United States accounted for 30% of the total publications for that time-period, Europe accounted for 23% and Japan publications accounted for 28%. World-wide (n = 178 patents, 1723 publications), United States (n = 159 patents, 393 publications), Japan (n = 217 patents, 562 publications), Europe (n = 67 patents, 267 publications), China (n = 98 patents, 49 publications), Canada (n = 13 patents, 12 publications).

To understand the distribution of effort in this field across the globe, each document was assigned a country. The documents were then aggregated based on location (Figure 5) to illustrate the contributions in both patents and publications from each region. The regions included in this analysis are the United States, Japan, Europe, China, and Canada. These countries were selected as they accounted for 88% of patents and 84% of publications that were identified prior to curation. To analyze the world-wide trends, we calculated the number of patents that were filed for coverage around the world and the sum of all publications regardless of origin. Each country that was investigated shows a rise in patent filings during the 2005 spike, with Japan making the largest contribution of 125 patents from 2001 to 2008. Since this surge in patents, Japan has maintained a low baseline number of 6 patents per year. The recent rise in patents is mostly attributed to the United States, China, and Worldwide filings. China has seen a particularly large increase in patent filings from 2017 to 2020, publishing 74 patents, which is more than triple the number of patents published since 1990.

The publication trends of the United States and Europe follow the global trend of a decrease in publications until about 2005 and then a continuous increase. Japan, however, showed the opposite trend where they begin with few publications and gradually built to 45 publications per year in 2010 before decreasing by an average of three publications per year. Chinese publications followed a similar trend as their patent filings. From 2018 to 2020, cell-free researchers in China nearly doubled all previous publications since 1990.

## 4. Discussion

Here, we analyzed the emergence of the cell-free synthetic biology field based on publication and patent frequency. The picture that emerges is that system development and applications are coming of age, with an increasing rate of research and potential. There are a few caveats in analyzing the progress and impact of a field using the patent and publication data. This study focuses on the total count of published patents and peer-reviewed manuscripts and their separation into various groups based on relevant metadata, such as their topic and authors. It does not investigate the long-term impact of these documents. Few highly influential advancements could impact the field far more than numerous others. We did not investigate the revenue generated by each patent, but our data suggest that various stakeholders were willing to invest the resources and effort necessary to file patents on the technology. It is also important to note that the observed changes in publications or patent filings are influenced by a variety of economic and societal factors that the current data set is unable to distinguish between, making the establishment of causal connections challenging.

Despite these caveats, we can make several conclusions. The number of patents and publications have been steadily increasing in cell-free synthetic biology over the last decade (Figure 2). Between 2015 and 2020, the most rapid growth in both patents and publications was related to improving CFPS systems (Figure 3). The constant growth in publications relates to applications of CFPS, which exceeded the other categories in recent years (Figure 3). In addition, there is growing commercial interest in CFPS technology by companies, as evident by numerous new start-ups in this space (Figure 4a). China has shown the most rapid growth in both publications and patents over the past few years, while Japan has shown diminishing numbers (Figure 5).

Based on these conclusions, we envision a few developments that could enhance investment and research interest in CFPS technology. Recent work has investigated the robust quality control of CFPS [50]. The focus on quality control will be critical to delivering cell-free products for practical use, especially in applications such as on-demand therapeutic production and point-of-care detection [21]. Furthermore, production yields of green fluorescent protein (GFP) are often used to benchmark the quality of CFPS, but GFP production does not reflect actual use cases. For instance, our recent work has benchmarked a new CFPS system using a range of proteins with different sizes, including a peptide nanocage, GFP, and Cas9 [51]. The creation of robust and multifunctional CFPS systems will also allow production of a broad range of proteins, varying in protein size, post-translational modification, and folding. This includes peptides and proteins with non-canonical amino acids [52,53,54,55]. In addition, the drastic reduction of the cost of CFPS systems and the ability to scale reactions will allow for the incorporation of these systems into routine lab workflows. Finally, the ability to readily store, distribute, and activate freeze-dried cell-free systems by simply adding water has opened new opportunities for on-demand biomanufacturing and point-of-care diagnostics [8,17]. Since CFPS is still less known and used in society, further development of educational kits [43,56,57] and courses will increase exposure and awareness of the technology to encourage the long-term expansion of the field.

## Data Availability

The complete set of patents and publications, including all metadata and categorization, is available in the Supplementary Dataset as an excel file.

## Supplementary Materials

The following are available online at https://www.mdpi.com/article/10.3390/life11060551/s1, Supplementary Dataset: Supplementary data file.
